# Experimental study and machine learning model to predict formability of magnesium alloy sheet

**DOI:** 10.12688/f1000research.124085.1

**Published:** 2022-09-29

**Authors:** Balaji Viswanadhapalli, Bupesh Raja V.K, Krishna Chythanya Nagaraju

**Affiliations:** 1 Mechanical Engineering, Gokaraju Rangaraju Institute of Engineering and Technology, Hyderabad, TELANGANA, 500090, India; 2 School of Mechanical Engineering, Sathyabama Institute of Science and Technology, Chennai, Tamilnadu, 600119, India; 3 Computer Science Engineering, Gokaraju Rangaraju Institute of Engineering and Technology, Hyderabad, TELANGANA, 500090, India

**Keywords:** Magnesium alloy sheet, Aerospace and automotive applications, Formability, Stretch forming, Forming Limit Diagram, Machine learning model.

## Abstract

**Background:** Magnesium alloy is not only light in weight but also possesses moderate strength. Magnesium AZ31-H24 alloy sheet has many applications in the automotive and aerospace industries. Experimental stretch forming tests are performed on this sheet to measure the material’s formability by constructing forming limit diagrams.

**Methods:** Several tests of Nakazima were carried out on rectangular samples at 24, 250, 350°C and 0.01, 0.001 mm/s using a hemispherical punch. The work done to predict the formability of magnesium alloys has not been recorded in recent literature on machine learning models. Hence, the researchers of this article choose to explore the same and build three models to predict the formability of magnesium alloy through Random Forest algorithm, Extreme Gradient Boosting, and Multiple linear Regression.

**Results: **The Random Forest showed high accuracy of 96% in prediction.

**Conclusions:** It is concluded that the need for physical experiments can be greatly minimized in formability studies by using machine learning concepts.

## Introduction

Magnesium alloy sheet in rolled condition, is an emerging alternative material to aluminium in light weight applications. Magnesium alloys having applications in the aerospace and automotive industries. ^1^ The low melting point of magnesium alloys as compared to steel alloys makes them suitable for casting. Standard automobile steel makeup is about 65% of its parts, which is important for safety reasons, but if the magnesium alloy is stronger and lighter the overall weight of automobile can be reduced to improve fuel efficiency. Fine-forming products can be produced with available metal-forming techniques to broaden the applications of magnesium alloys. In the past, due to the low ductility of magnesium sheets, the applications are limited. However, it was of particular interest because its low density could result in savings in vehicle weight, in turn fuel economy which is a major concern in the automotive and aerospace industries. Hot working can improve the ductility of the material when formed at elevated temperatures. The basal slip mechanism and twinning are the major deformation mechanisms. At temperatures of 200°C and above, some of these basal planes can get activated. ^2^

The new version of the AZ31B magnesium alloy AZ31B-H24 was used in this present work. Formability, however, is limited by several factors such as poor elongation, alloying elements, and mainly by forming speed and temperature.

### Plastic deformation and magnesium alloy

The key to effectively use light alloys like magnesium lies in understanding how they are structured at the atomic level. Magnesium, like Titanium, has a hexagonal close-packed (hcp) crystal lattice with a packing factor of 74%. These lattices are not perfect, but they have many defects such as spacing and linear defects. ^3^ Linear defects such as edge dislocation and screw dislocation are important in metal forming processes. Dislocations mean that the number of atoms are offset from their normal position in the lattice. Edge dislocations have an additional half-plane of atoms that, when stress is applied to the lattice, the atomic bonds are broken and re-formed, allowing the additional half-plane atoms to glide through the lattice. Screw-dislocation, on the other hand, occurs when the entire block of atoms is shifted out of alignment with the correct lattice structure when shear stress is applied to it.

**Figure 1.**
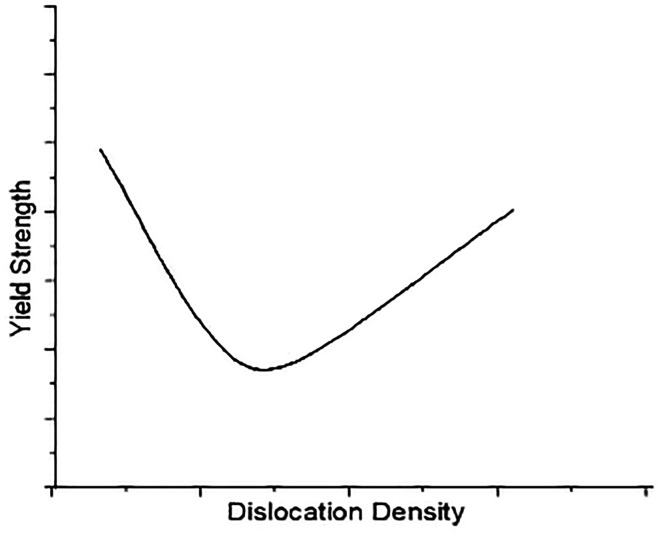
Influence of dislocation density on Yield strength.

**Figure 2.**
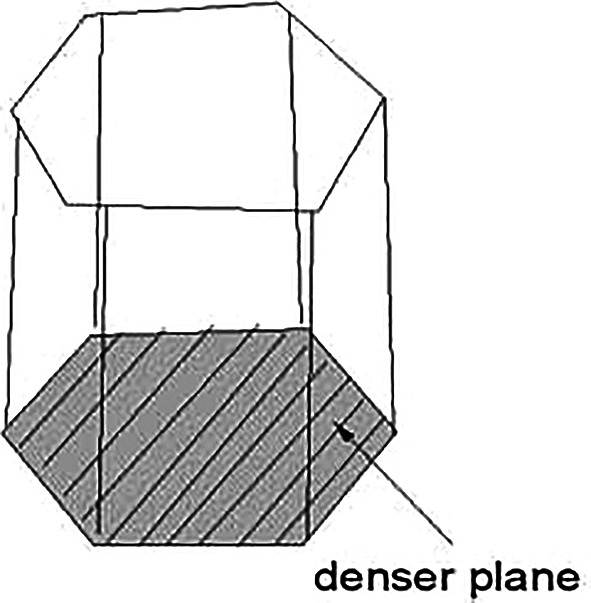
Denser plane in hcp structure.

This displacement motion is irreversible when the tension is removed. The main concept behind plastic deformation is the movement of many dislocations at the atomic level. Material yield strength varies with dislocation density. Materials that contain many dislocations have improved strength, preventing each other from moving through the lattice. Figure 1 shows the effect of dislocation density on the material yield strength. The movement of dislocations through the lattice also affected by how the atoms are packed together. It is easiest for dislocations to move along the planes where the atoms are closest to each other which are the most densely packed planes because those bonds are easier to break and re-form. The denser plane in hcp is shown in Figure 2. ^4^

### As received AZ31B-H24 magnesium alloy sheet

In AZ31B-H24 magnesium alloy, H24 indicates one of the tempered designations. H24 means strain hardened and partially annealed. ^5^ The molten metal moves along the channels into moulds to form huge slabs of magnesium, which in turn will roll the solid slab into sheets. From time to time a small sample of molten magnesium is taken for analysis. The metal is poured and left to solidify. As magnesium solidifies, tiny metal crystals begin to form and grow at several points in the liquid in a special film. Each crystal moves outward in all directions until it meets the surfaces of its neighbouring crystals. In engineering parlance, each fully grown crystal is called a grain. Once the magnesium is poured into the slab it is rolled into sheets. To reduce the metal to the desired thickness, it is now rolled several times while relatively cold, as the metal is crushed between the rollers as it is cold worked. Cold working influences the grain structure of magnesium. Changes in grain structure by cold working are accompanied by changes in the mechanical properties of the metal. Its hardness and tensile strength increase while ductility decreases. After cold working the metal is usually heated to a sufficiently high temperature. Heating influences the deformed grain structure. Nothing happens until the metal is heated to its recrystallization temperature, but after attaining its recrystallization temperature, new grains begin to grow rapidly at the grain boundaries and until a new identical grain is formed. The structure does not completely replace the old deformed one. This process is known as recrystallization. This influenced the mechanical properties. These newly formed grains are separated by grain boundaries. Since each grain has a specific plane along which it is easy to slip. ^6^ The presence of grains impedes the movement of dislocations, so polycrystalline metals are stronger than metals composed of similar crystals. The smaller the grain size, the stronger the material.

All metals and metal alloys are composed of grains, although the grains are not visible. To make the grain visible, it’s a common practice to a give firstly a mirror-like finish and then carefully treating the finished surface with a very strong acid. ^7^ Gloves are required for this operation. As soon as the acid has time to react, the metal must be washed. It is then given a second treatment with another special chemical, this process commonly called as etching. The chemical used for etching is dependent on the material. At the last wash, there are granules of similar size and shape. But in structure they appear as different shapes only because they reflect light. Etching is an important process as it reveals the grain structure. In this present work is AZ31B-H24 magnesium alloy, which is partially annealed, and strain hardened. Thus, the metal is softened and prepared for further work such as shaping, stamping, or forming. AZ31B magnesium alloys have major advantages such as good ductility and corrosive resistance. ^8^ Good strength, high electrical and thermal conductivity at room temperature. AZ31B-H24 magnesium alloy is a newer version of AZ31B magnesium alloy, which has applications in the production of complex parts, chassis components and structural engine parts, etc., where low weight and high relative strength are required. It’s observed that the resistance to indentation on a piece of metal before and after recrystallization, recrystallization has restored softness which means a reduction in hardness. And the tensile strength of the recrystallized magnesium sample was also deceased when tested compared to the cold worked sample. It is noteworthy that the recrystallized sample is most stretched. Hence restored the flexibility. However, the resulting properties depend on the temperature at which recrystallization is performed. If the temperature is too high some grains will grow at the expense of their neighbours. This can lead to properties that are highly undesirable for most engineering applications. ^9^

### Experimentation-stretch forming

**Figure 3.**
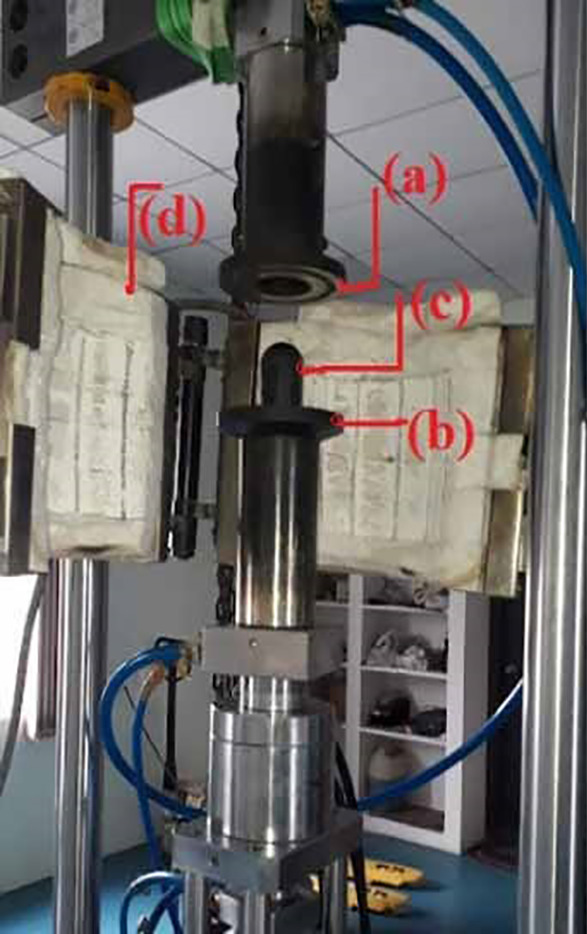
Hot forming equipment for stretching operation with major parts (a) Upper blank holding die; (b) Lower blank holding die; (c) Hemispherical punch; (d) Insulated heating chamber.

Bi-axial stretch forming test was performed on AZ31B-H24 magnesium alloy of 2 mm thick sheet. ^10^ Rectangular specimens of different widths of 150 mm, 125 mm, 100 mm, and 75 mm are taken, fixing the other dimension as constant at 150 mm. However, other smaller widths such as 50 mm and 25 mm were not taken, as this work mainly focuses on biaxial stretching and plane strain conditions. CNC wire cut edm process has been preferred to cut the samples to the desired sizes. Shearing process, is not preferred for preparing the samples, as it associated with high cutting forces, thereby resulting reduced formability. To measure the deformations, a circular grid is etched on the samples with a laser engraving machine prior to stretching. The circles can take the shape of ellipses after being stretched. The major and minor diameters of the ellipses are measured to calculate the strains and predict the forming limits of the material. No lubricant is applied on the sample in this work. However, lubricant may have a marginal effect on formability of sheet metal. ^11^ Many Nakazima tests are conducted using hemi-spherical punch of 50mm diameter. Hot forming equipment have been used in this work was shown in Figure 3. The equipment is connected to computer data acquisition system to interpret the data. Specimen is heated to reach desired temperature.

A soaking time of 20-30 min is allowed as per standards for test specimen to attain equilibrium temperature inside the heating chamber. For this purpose, ceramic wool is used as insulating material to avoid heat losses from the heating chamber. An initial blank holder pressure of 120-140 bar is applied on the specimen by using a hydraulic pump with 300 bar capacity. ^12^ The blank holder pressure essentially creates a bead on the flat sheet prior to start of actual stretch forming operation using upper and lower dies. The experiment is monitored by observing the load -displacement curve displayed on the computer screen via data acquisition system.

**Figure 4.**
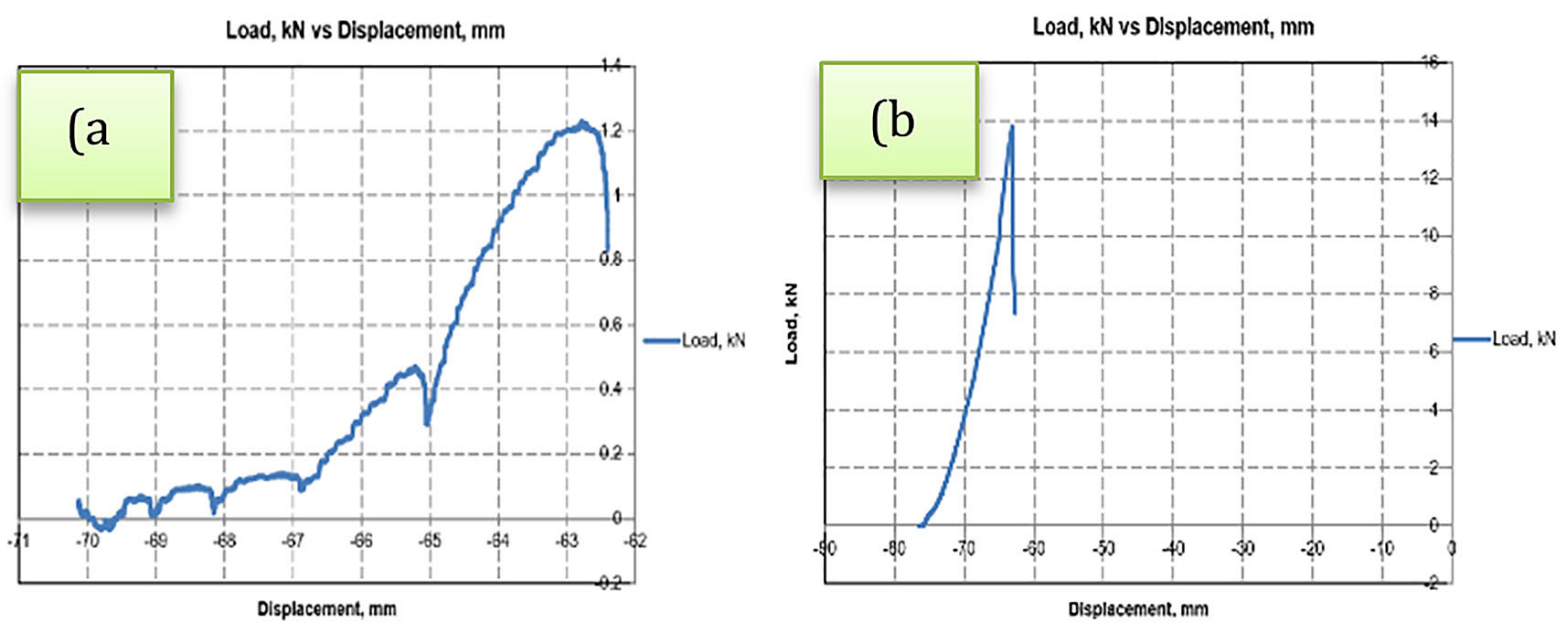
Load-Displacement curve depicting (a) Several Yielding points at 350°C, and (b) No Yield point at room temperature.

**Figure 5.**
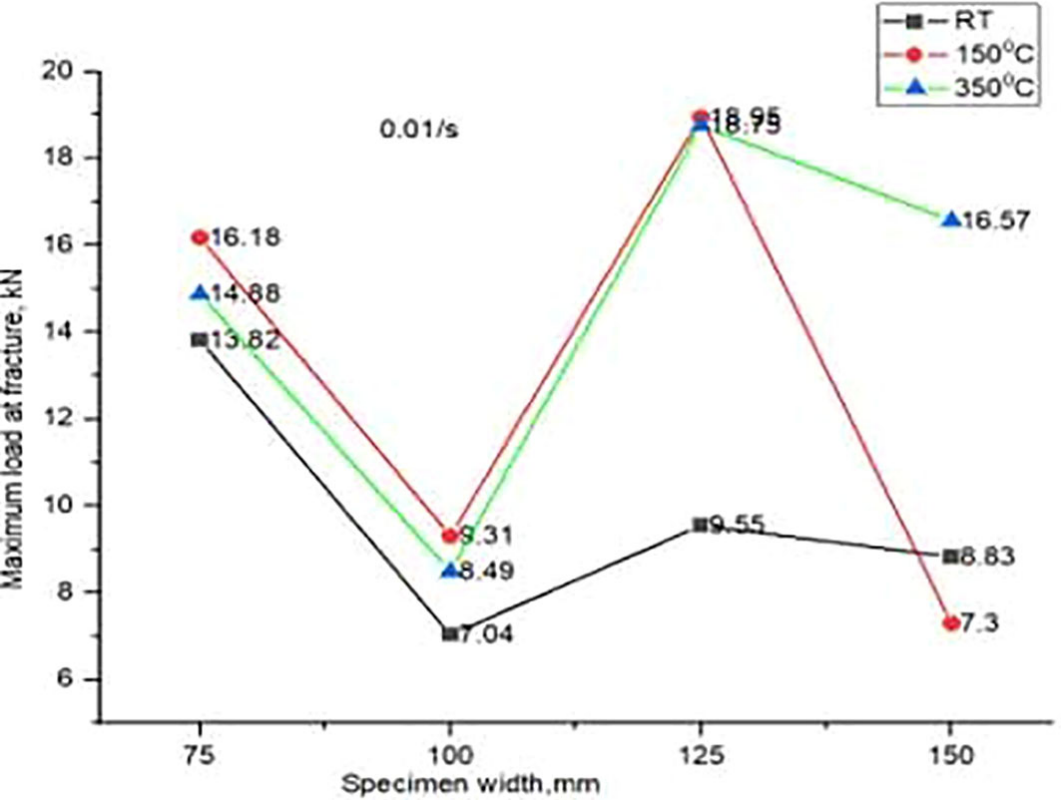
Maximum load carrying capacity of four different specimens formed at one fixed strain rate and 3 different temperatures.

It’s a challenging task to predict the exact moment and time the fracture begins. It is common practice to stop the experiment by observing the load-displacement curve displayed on the monitor. Many yielding-like points are observed while stretched at temperatures 250°C and above and no such points appear when stretched at room temperature as shown in Figure 4. Reason behind these yielding points has yet to be investigated. Maximum load at fracture for different temperatures, at one fixed strain rate, on four different widths have been noted from the experiments and are analysed as shown in Figure 5.

At 150°C and 0.01mm/s, it is observed that maximum dome height and maximum load at fracture. However, specimen width has marginal effect on maximum load at fracture. Temperature and strain rate has got more impact on load carrying capacity. Temperature and speed are the two dominant parameters when compared to other parameters like width of the specimen, thickness, and lubrication. Blank holder pressure can be adjusted, otherwise would cause crack initiation near the bead even before the start of forming process.

**Figure 6.**
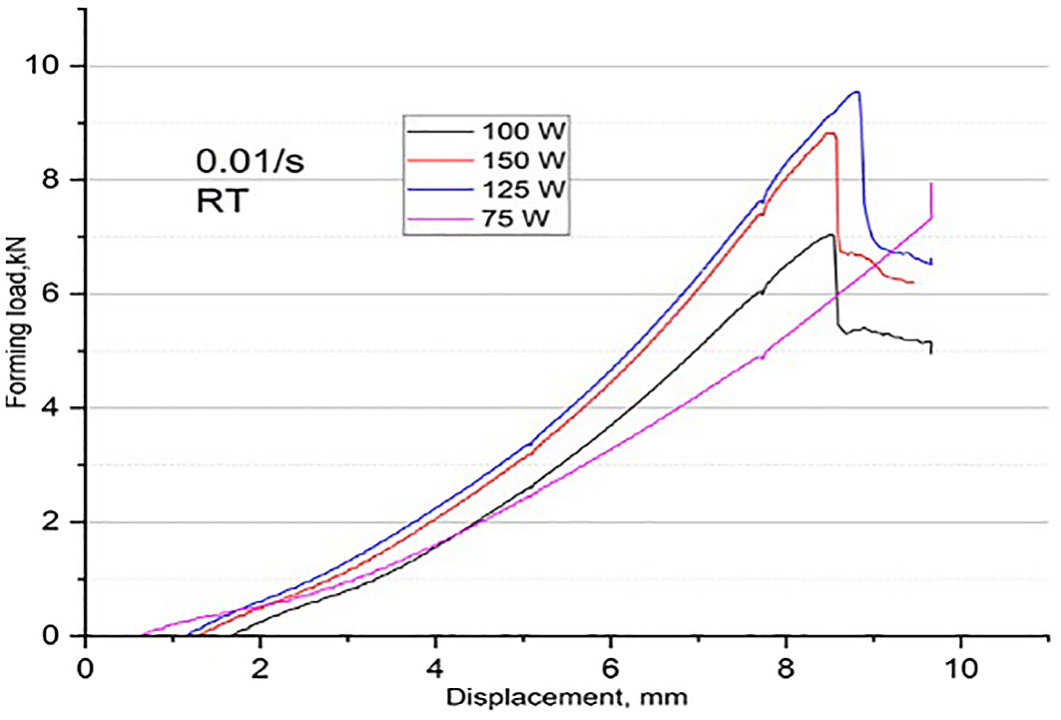
Forming load Vs forming displacement is plotted.

**Figure 7.**
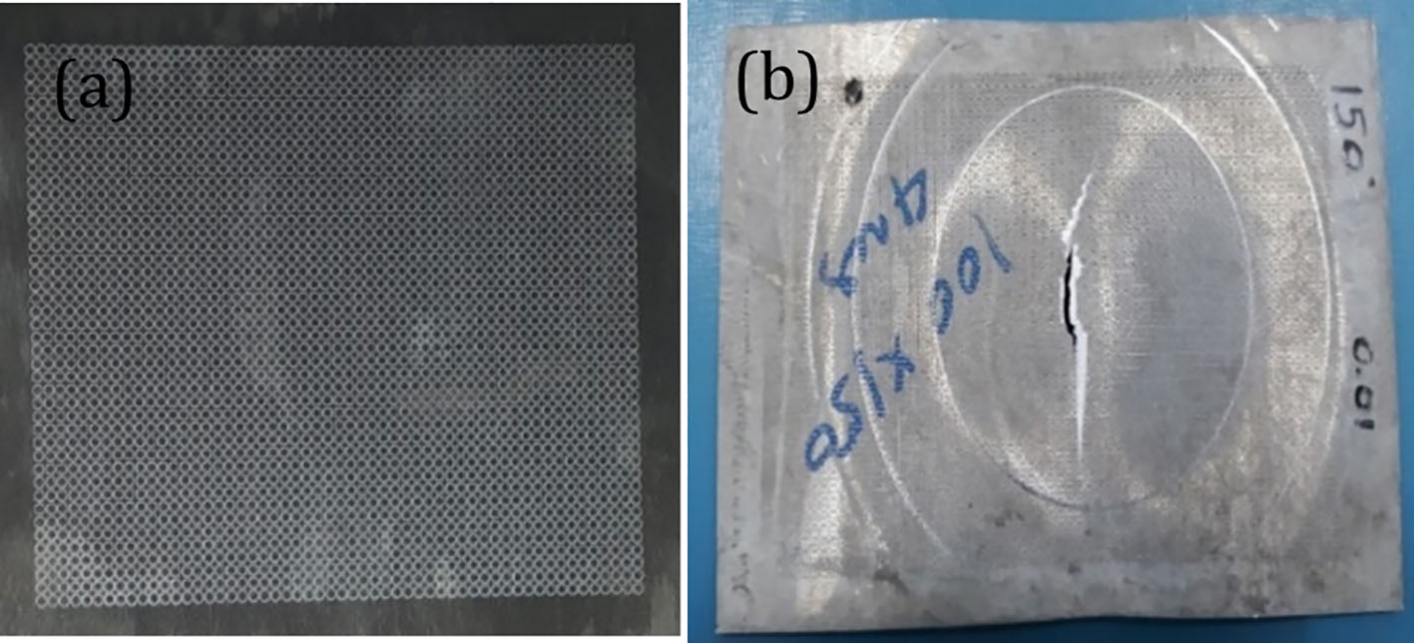
Magnesium sheets (a) magnesium etched specimen; (b) specimen with bead formation and necking after stretch forming operation.

**Figure 8.**
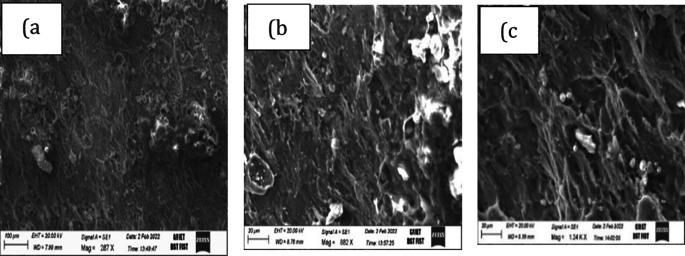
Fractographs of magnesium alloy exhibiting ductile fracture when formed at 150°C at 0.01 mm/s at different magnifications (a) 287x (b) 882x and (c) 1.43kx.

**Figure 9.**
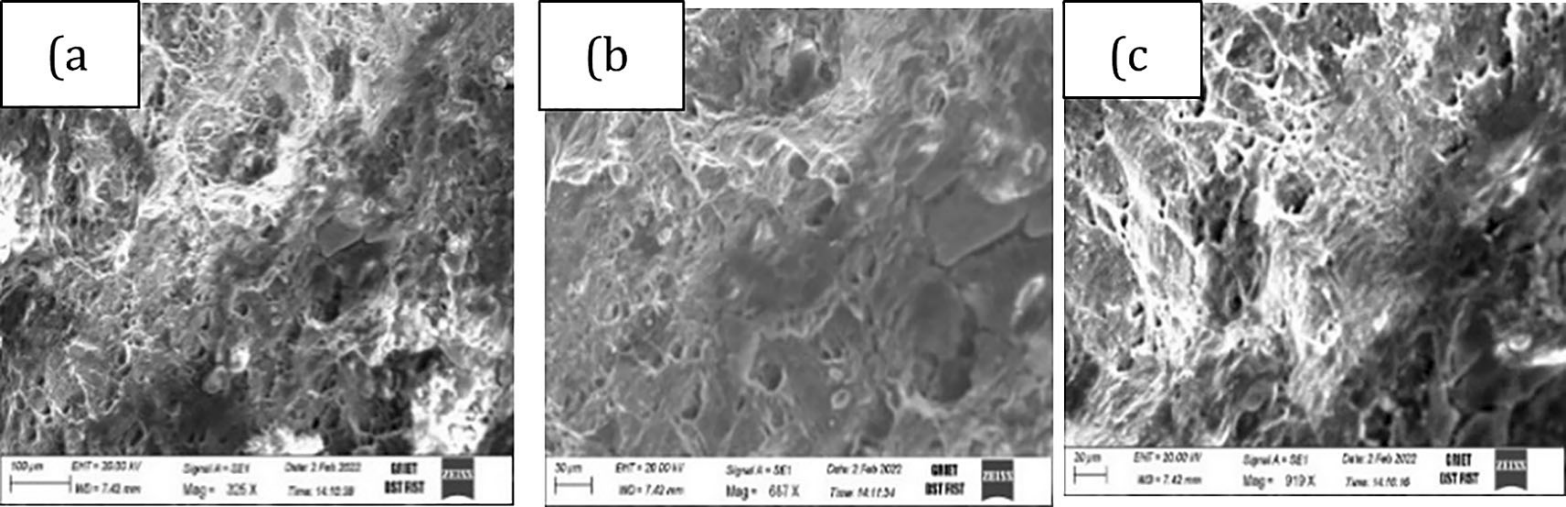
Fractographs of magnesium alloy when formed at 350°C at 0.01 mm/s at different magnifications (a) 325x (b) 687x and (c) 919x.

To determine the formability of the material, forming limit diagrams are constructed by measuring the circle dimensions before and after the forming operation From Figure 6, it is clearly observed that the load-displacement curves are almost equal irrespective of specimen widths at room temperature and at other temperatures as well. ^13^ At 0.01/s and RT, load and displacement peaks are much less observed compared to the highest values obtained when stretching at 0.01/s and 150°C. It is observed that even at 350°C, the maximum load on the fracture is much lower if the rate of formation is 0.001/s. Therefore, the impact of forming speed cannot be ignored. Figure 7 shows a magnesium etched specimen and another specimen with clear beading and necking after stretching. Fractography, a failure analysis method to study the fracture surface of materials. Studying fracture surface characteristics can help determine the cause of failure in an engineered product. Specific failure modes impart characteristic features on the fracture surface. A small piece of cut sample is taken at the fracture zone with CNC wire cut edm and tested for fractography. Scanning electron microscope pictures are shown in Figure 8 and Figure 9. Both the fractographs exhibiting the ductile fracture, which is associated with dimples and inclusions, a favourable condition for good formable material. Crack initiation is so gradual in ductile fracture.

### Construction of forming limit diagram

Any information related to a certain material can be obtained by an experimental tensile test. The Ultimate Tensile Strength as an example at which the necking occurs, cannot be used as a single failure criterion. Instead, forming limit diagram (FLD), used to predict the forming behaviour of sheet metal. ^14^ The forming limit curve is a material parameter that reflects the limiting strains that cause necking failure as a function of the strain path. It is a function of the quality of the metal, the thickness and surface conditions of the sheet, as well as the methods used during its creation vide hemispherical or flat punch, test speed, temperature. It is applicable to any shape of part.

**Figure 10.**
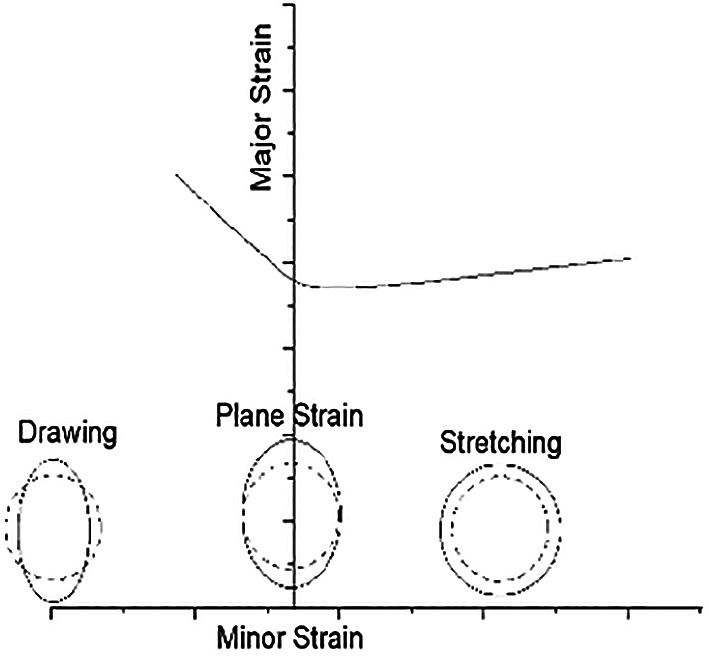
Typical forming limit diagram illustrating various modes of failure strain conditions.

**Figure 11.**
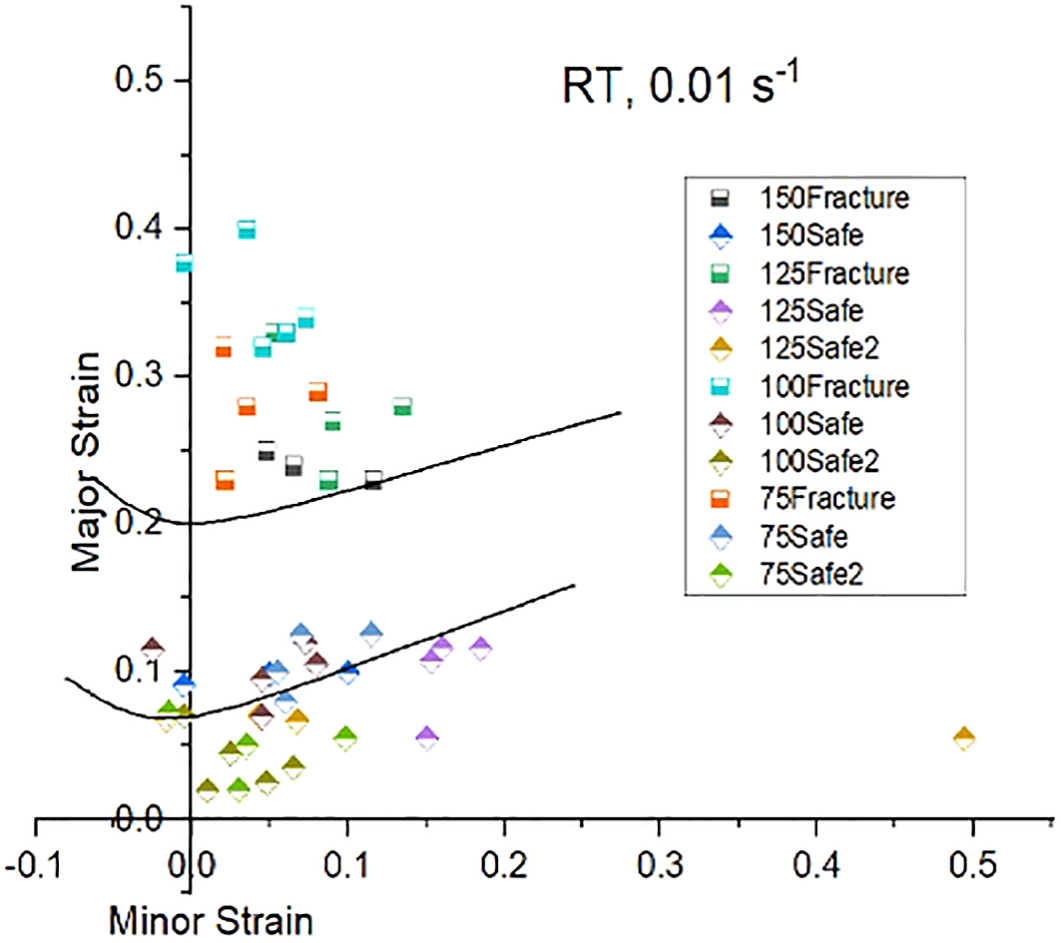
Experimental Forming Limit Diagram showing Forming Limit Curve (top) and Marginal safe curve (bottom) when stretched at sheet temperature 24°C and 0.01 mm/s.

**Figure 12.**
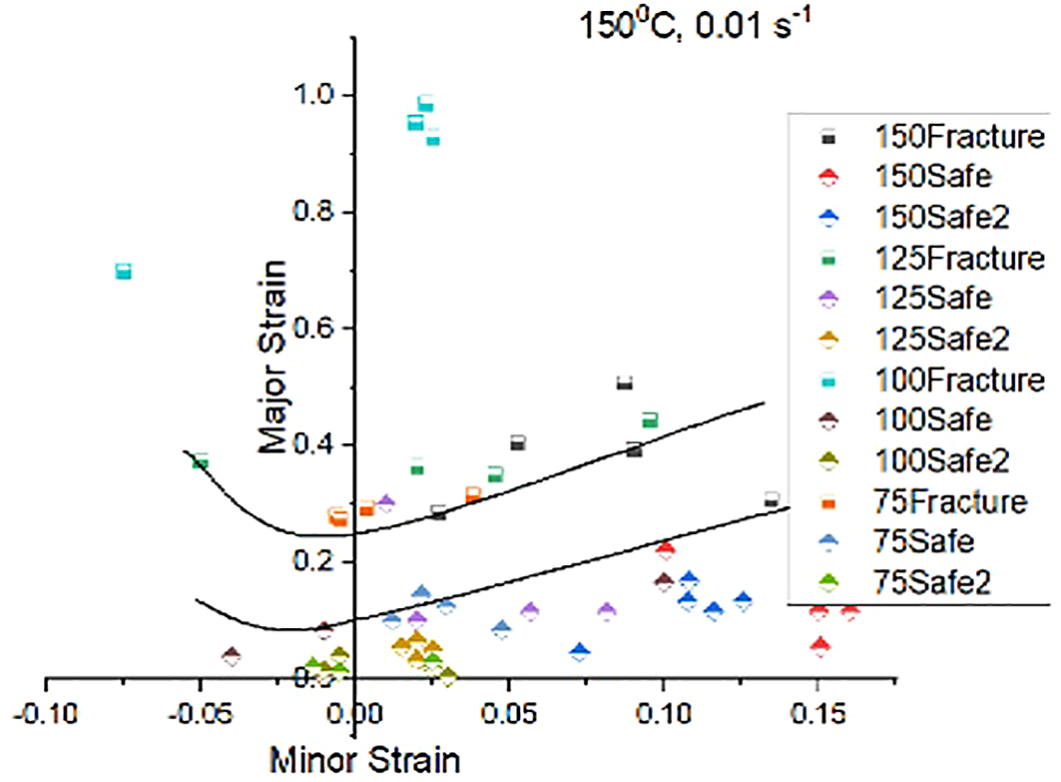
Experimental Forming Limit Diagram showing Forming Limit Curve (top) and Marginal safe curve (bottom) when stretched at test temperature 150°C and strain rate of 0.01 mm/s.

**Figure 13.**
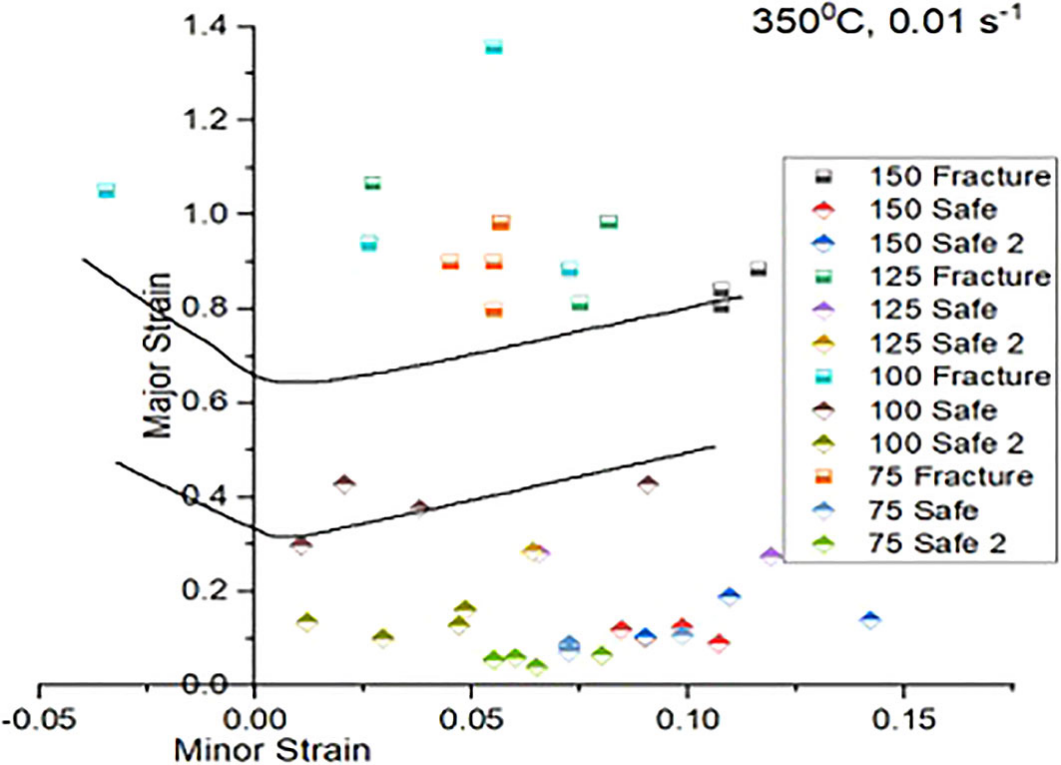
Experimental Forming Limit Diagram showing Forming Limit Curve (top) and Marginal safe curve (bottom) when stretched at sheet temperature 350°C and punch speed of 0.01 mm/s.

FLD is a diagram contains major principal strain values that are always positive and minor principal strains that are either positive or negative. The shape of the curve can be obtained experimentally. To construct forming limit curve, test pieces of different geometry stretched by a punch with a diameter of 50 mm until fracture occurs. It is a regular practice to make a circular grid all over the structure or panel and after the metal forming process is carried out to see or monitor the behaviour of the circle at each region on the specimen panel. Up to 4 or 5 Nakazima tests can be performed using each of the specimens for the average point on the plot. It is observed that some regions are uniaxial state of stress, some regions are of biaxial state of stress and some other regions are of plane-strain condition as shown in Figure 10. Experimental forming limit diagrams have been plotted by considering 4 or 5 ellipses on either side of the necking zone from each specimen. Stereozoom optical microscope is used to measure major and minor diameters, which will be useful in calculating major and minor true strains. Figure 11, Figure 12, and Figure 13 shows experimental FLDs, when stretched at room, 150°C and 350°C temperatures and at strain rate 0.01/s respectively.

### Machine learning models to predict formability

Having proven itself a better option for prediction in the fields of Finance, Natural Language Processing, weather forecasting, computer vision etc Machine Learning has slowly paved its path into Material Science as well in the recent years. With the advent of machine learning models and artificial intelligence once again gaining much ground in the research front, much of work is being carried out in developing machine learning models to study different alloy properties. The machine learning mechanisms is largely dependent on the data set used to train the model. The required features to calculate or measure a specific feature of the alloy are considered as the input set of features and the output variable is the feature in interest to be calculated. The model builds up a quantitative relationship between the input features and the target variable. Zhenxin Lu, in his work, predicted successfully the corrosivity of low alloy steel. ^15^

In the recent past many researchers have given their contributions in developing machine learning models for prediction of metallic materials’ properties. D.Z. Xue studied accelerated search for materials with targeted properties by adaptive design. ^16^ C. Wen studied Machine learning assisted design of high entropy alloys with large hardness. ^17^ S. Feng identified the defects in stainless steel using deep neural network. ^18^ Y.T. Sun carried out Prediction of glass forming ability in binary metallic alloys using machine learning approach. ^19^ C. Wolverton in his studies used machine learning techniques to study Accelerating design of engineered metallic glasses. ^20^ The study done by L W Hart, is an excellent work of review on current research happening on alloys based on Machine Learning. ^21^ In the work carried out by Amar M Chheda, a ML model was developed to predict the forming limit diagrams of aluminium alloys. ^22^ The researchers initially considered multi-dimensional data but after rigorous data reduction mechanism application and elimination of non-importance data in recursive method, it was concluded to use 12 features to train the ML Model. Out of 12 features ‘n’and ‘r’ values from tensile tests were used besides elemental composition being represented by six features and four parameters representing process. Accuracy calculation was done based on Root mean Square error ration with respect to R ^2^ and a global genetic algorithm was used for fine tuning of parameters with leave-one-out cross validation score being used to calculate accuracy of model. The researchers claim to have around 93% accuracy. The study carried out by Zhenxin Lu, tried to predict the corrosion potential of magnesium alloy based on its chemical composition. The study was based on four machine learning models developed using Extreme Gradient Boosting, Random Forest, Support Vector Machine regression and Multiple linear regression. The work of Leyun Wang, builds two models based on Artificial Neural Networks and Support Vector Machine algorithms to predict the tensile properties of AZ31 Magnesium alloy. ^23^ Tensile strength, Yield Strength and Ultimate Tensile Strength were predicted using above models. But, the models could not accurately predict the Elongation. The work made use of 112 data items. The authors considered 16 features as input for ANN model. However, the work carried to predict the formability of magnesium alloy was not recorded in the recent literature of Machine learning models being developed to study the alloy properties. Hence, the researchers of this article choose to explore the same and build a model to predict the formability of Magnesium alloy using Random Forest algorithm.

### Machine learning model set-up

**Figure 14.**
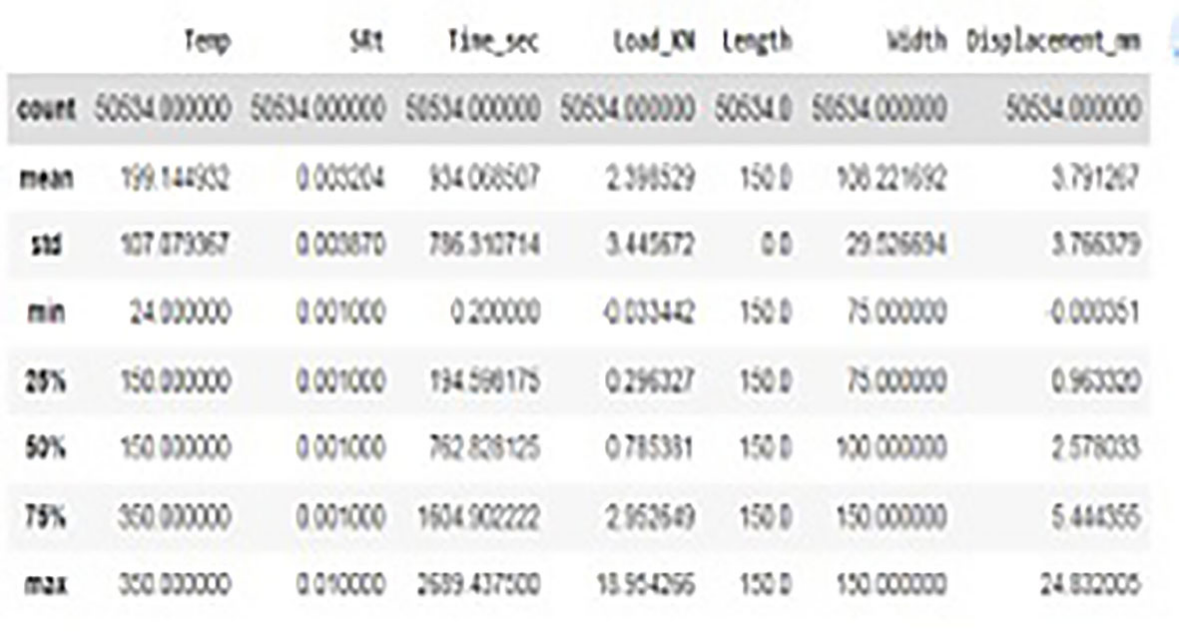
Description of Data set.

**Figure 15.**
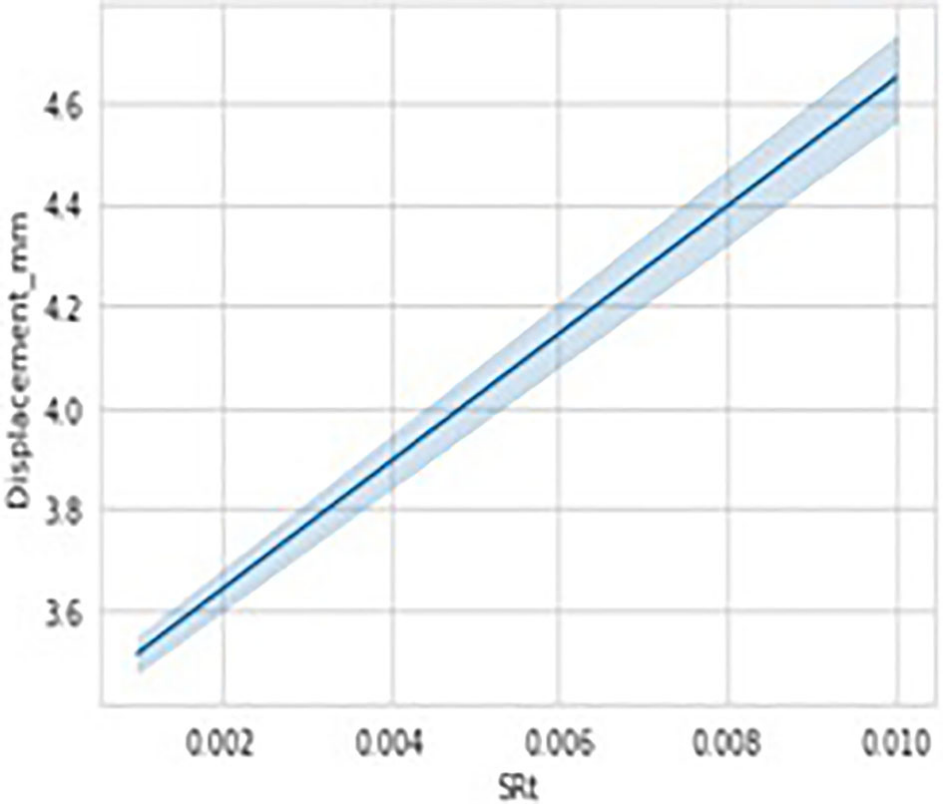
Data visualisations-stain rate vs displacement.

**Figure 16.**
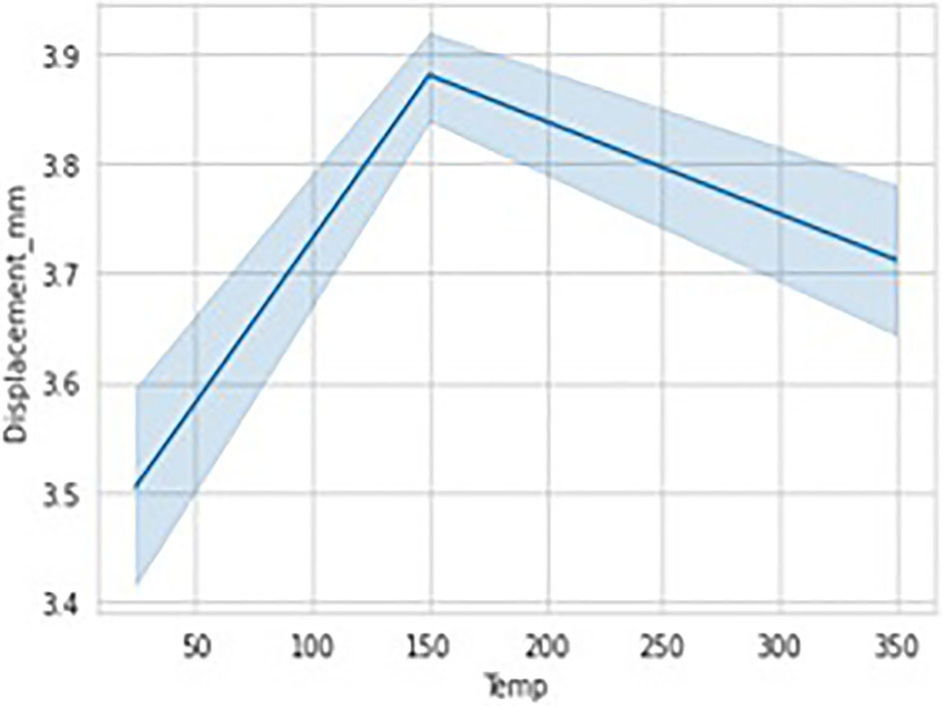
Data visualisations-temperature vs displacement.

**Figure 17.**
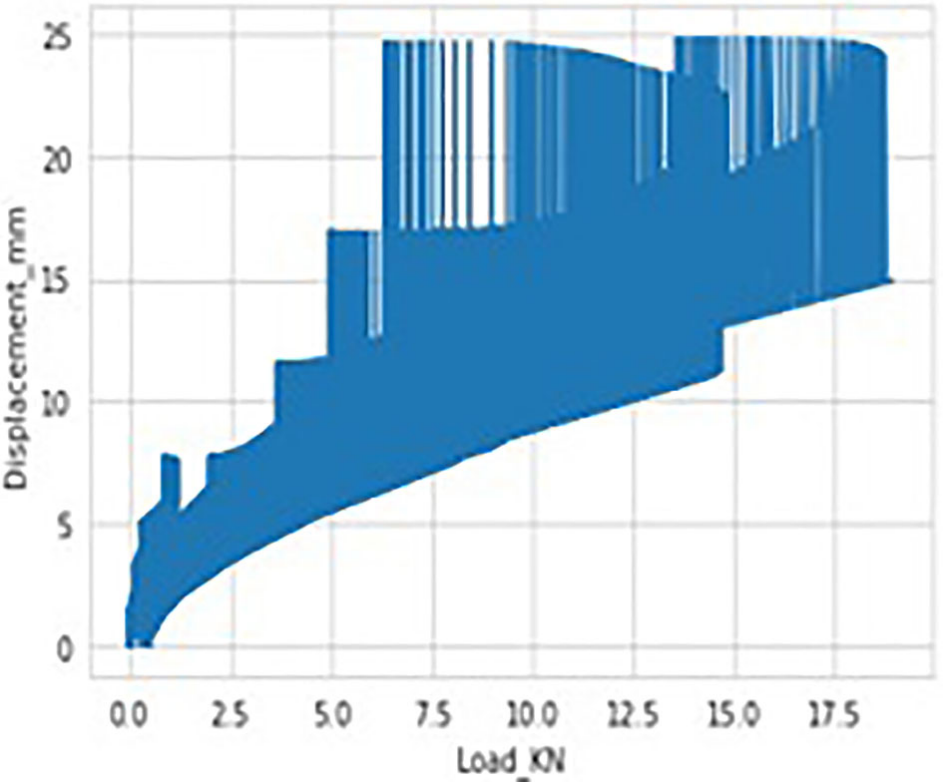
Data visualisations-load vs displacement.

The data set is of 50534 rows and 7 columns. We used Temperature, Stain Rate, Time in Sec, Load in kN, Length, Width properties of experiment as input features for training and Displacement in mm as target variable. The description of the data can be found as shown in Figure 14. The data considered has got different temperatures viz, 24 (room), 150 and 350 degrees respectively and the Stain Rate (SRt) values are 0.01 and 0.001 mm/s. As it can be observed there were no data missing cases as we can observe each column having equal count of 5034 values. Hence, we can conclude there are no anomalies in data considered. The data visualization graphs that are Strain rate Vs displacement, Temperature Vs Displacement and load Vs displacement illustrated in Figure 15 and Figure 16, and Figure 17 respectively, to understand and interpret the data more clearly.

**Figure 18.**
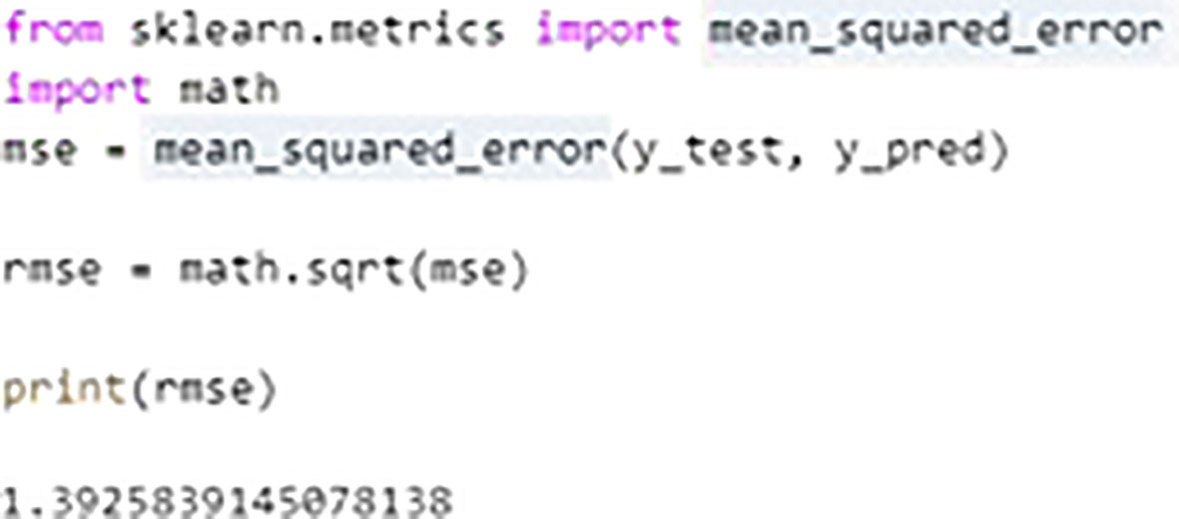
RMSE value of multiple linear regression.

**Figure 19.**
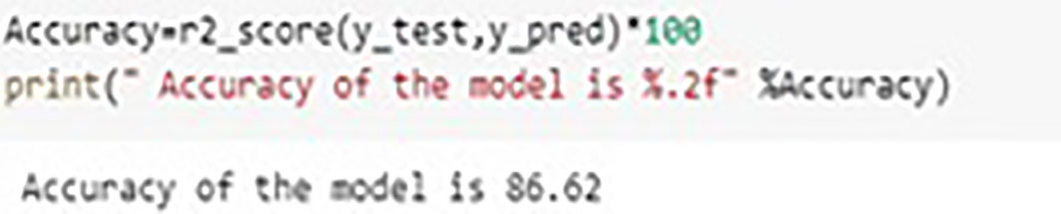
Accuracy of MLR.

**Figure 20.**
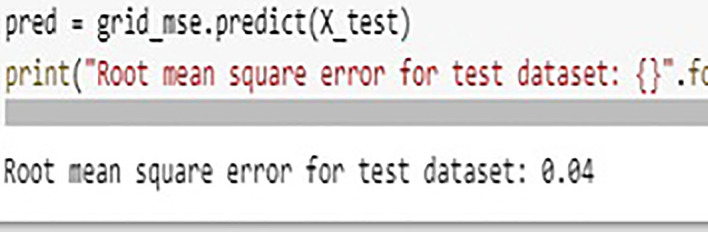
RMSE in XGBoost.

**Figure 21.**
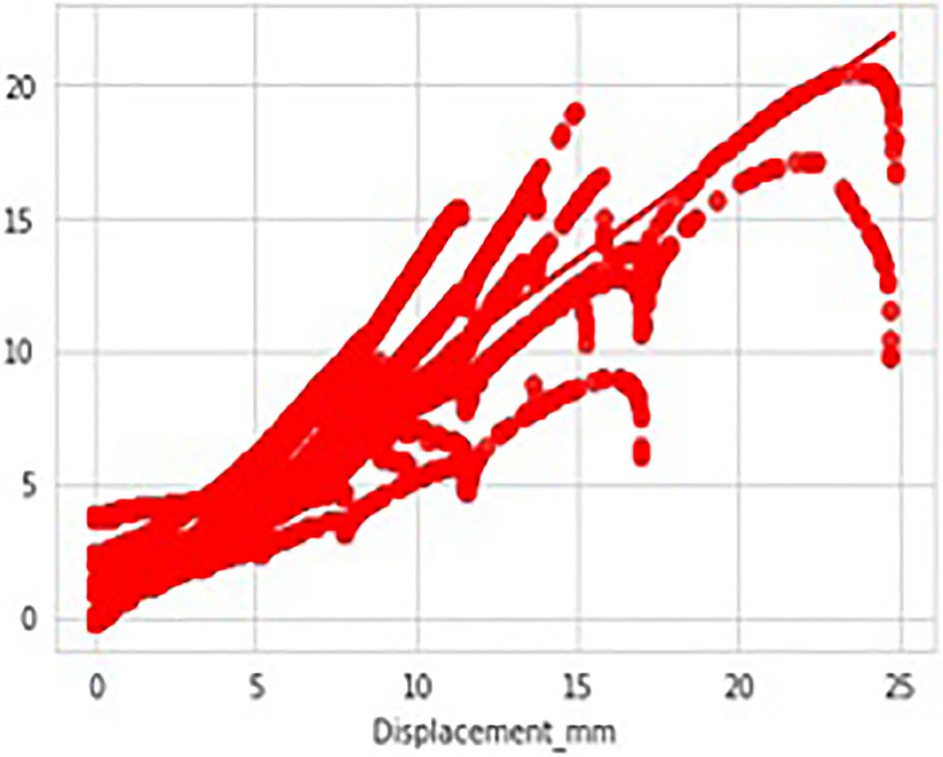
The regression plot in MLR.

The Random Forest builds multiple Decision Trees and the output of multiple trees is averaged to get the final output of Random Forest. Random Forest is categorized under Supervised learning techniques of Machine Learning. The decision trees are evaluated parallel and it is also called as Bagging technique. Owing to the feature and subset randomization, this technique of random forest almost avoids over fitting of data. In this work authors explored three different machine learning models of Random Forest (RF), Extreme Gradient Boosting (XGBoost), and Multiple Linear Regression (MLR). It was observed that the accuracy in RF was almost 94% and in case of EGBoost Root Mean Square Error (RMSE) was just 0.04 whereas in case of MLR it was observed that the accuracy is of only 86.62% and RMSE was 1.392, thus RF demonstrated better performance in predicting the formability of Magnesium Alloy in our experimentation. The graphs shown in Figure 18, Figure 19 and Figure 20 indicating the values, accuracy and RMSE of different algorithms. The regression plot for displacement in MLR is shown in Figure 21.

## Results and discussion

Stretch forming experiments were conducted on 2mm thick AZ31B-H24 magnesium alloy sheets to find material’s formability using hemi spherical punch at elevated temperatures.

For this purpose, many Nakazima tests are performed on each specimen for consistency. FLDs are constructed, which is the measure of formability.

Using the experimental data set, ^24^ three Machine learning models are developed to predict the material’s formability. Results and observations from both experimental study and machine learning models are summarised below:

## Experimental results

• Maximum height of the dome is achieved when formed at test temperatures of 150°C and at strain rate of 0.01 mm/s.
• It is observed that the blank holder pressure of 120-140 bar can be successfully applied to the specimen, to create a good bead and to avoid cracks near the bead prior to forming happens.
• Punch speed of 0.01/s and blank temperature of 150°C have been identified as excellent forming conditions.
• Forming limit curve reveals the fact that the effect of sample temperature and strain rates are the major controlling factors for a good formability.
• Scanning electron microscope images indicate the ductile fracture, a favourable condition for good formability.

### Machine learning model results

• In predicting material’s formability, Random Forest (RF) model shown almost 94% accuracy.
• In case of XGBoost Root Mean Square Error (RMSE) was just 0.04.
• It is observed that the accuracy in case of MLR is of only 86.62 and RMSE was 1.392
• RF demonstrated better performance in predicting the material’s formability

## Conclusion

High temperature stretch forming tests are conducted on AZ31B-H24 magnesium alloy and the test results are used in developing and train the Machine learning models. The FLD curves and SEM images illustrated, demonstrating the fact that material’s formability can be significantly improved at high test temperatures, making suitable for temperature applications.

Machine learning models are built to predict the material’s formability. The three algorithms predicting materials properties accurately, hence the requirement of physical experimentations can be greatly avoided, thereby, provide emission free environment, if we can use light magnesium alloy in aerospace and automotive applications.

## Data availability

### Underlying data

Figshare:Stretch Forming _Data_Set, https://doi.org/10.6084/m9.figshare.20237529.v11. ^24^

This project contains the following underlying data:

- Temp_room_Strainrate_0.01_Samplesize_150X100mm.xls
- Temp_150_Strainrate_0.001_Samplesize_150X75mm.xls
- Temp_150deg_Strainrate_0.001_Samplesize_150X100mm.xls
- Temp_150deg_Strainrate_0.01_Samplesize_150X125mm.xls
- Temp_350deg_Strainrate_0.01_Samplesize_150X75mm.xls
- Temp_350deg_Strainrate_0.01_Samplesize_150X150mm.xls
- Temp_350deg_Strainrate_0.01_Samplesize_150x125mm.xls
- Temp_room_Strainrate_0.01_Samplesize_150X150mm.xls
- Temp_room_Strainrate_0.01_Samplesize_150X75mm.xls
- Temp_room_Strainrate_0.01_Samplesize_150X125mm.xls
- Temp_150deg_Strainrate_0.01_Samplesize_150X75mm.xls
- Temp_150deg_Strainrate_0.01_Samplesize_150X100mm.xls

Data are available under the terms of the Creative Commons Attribution 4.0 International license (CC-BY 4.0).
