# Supplementary material for: Experimental study and machine learning model to predict formability of magnesium alloy sheet
Source: F1000Res. 2022 Sep 29;11:1118. [Version 1] doi: 10.12688/f1000research.124085.1 (PMC10457562; doi:10.12688/f1000research.124085.1)
Supplement: Peer review history

# Peer review: Reviewer response for version 1

**DOI:** 10.5256/f1000research.136256.r193629

**Published:** 2023-08-25

**Authors:** Umer Masood Chaudry

**Affiliations:** 1 Department of Mechanical Engineering, Sungkyunkwan University, Jongno-gu, Seoul, South Korea

The present research utilized ML to predict the formability of AZ31-H24 Mg alloy. Overall, the manuscript looks interesting and can be Approved after addressing the following comments:

1. There are many grammatical mistakes throughout the manuscript. 
2. The quality of most of the images is very poor.
3. For the predicted data set, it would be better to show the graph with experimental and predicted formability comparison. 
4. More details about the algorithm used in the present study would be better for the reader.
5. Authors are suggested to add important literature about the mechanism controlling the formability of Mg alloys. A few suggestions are: Chaudry, Hamad & Kim (2019) ^1^, Chaudry, Kim & Hamad (2019) ^2^ and Masood Chaudry *et al.,* (2022) ^3^.

Is the work clearly and accurately presented and does it cite the current literature?

Partly

If applicable, is the statistical analysis and its interpretation appropriate?

Yes

Are all the source data underlying the results available to ensure full reproducibility?

Partly

Is the study design appropriate and is the work technically sound?

Yes

Are the conclusions drawn adequately supported by the results?

Yes

Are sufficient details of methods and analysis provided to allow replication by others?

Partly

Reviewer Expertise:

Plastic deformation of Mg, physical metallurgy, Materials for extreme environments

I confirm that I have read this submission and believe that I have an appropriate level of expertise to confirm that it is of an acceptable scientific standard.

## References

[1] : On the ductility of magnesium based materials: A mini review. *Journal of Alloys and Compounds* .2019;792: 10.1016/j.jallcom.2019.04.031 652-664 10.1016/j.jallcom.2019.04.031

[2] : Effect of Ca addition on the room-temperature formability of AZ31 magnesium alloy. *Materials Letters* .2019;238: 10.1016/j.matlet.2018.12.013 305-308 10.1016/j.matlet.2018.12.013

[3] : Designing highly ductile magnesium alloys: current status and future challenges. *Critical Reviews in Solid State and Materials Sciences* .2022;47(2) : 10.1080/10408436.2021.1947185 194-281 10.1080/10408436.2021.1947185

# Peer review: Reviewer response for version 1

**DOI:** 10.5256/f1000research.136256.r193630

**Published:** 2023-08-24

**Authors:** Kun Li

**Affiliations:** 1 Chongqing University, Chongqing, Chongqing, China

In this research, high-temperature tensile forming tests were conducted on AZ 31 B-H24 magnesium alloy and the results were used to develop and train machine learning models. Three models were developed to predict the formability of magnesium alloys by RF, XGBoost and MLR. There are some major suggestions for this article as follows:

**Comment 1: **The paper lacks a reasonable explanation for the utilization of three machine learning methods.

**Comment 2:** The explanations of the diagrams in the text are too little explained, please add them. For example, Figures 4, 8, and 9.

**Comment 3:** The quality of the diagrams in the paper is terrible. It is not very impressive in terms of conveying what is stated as well as the clarity of the picture. Please replace it.

**Comment 4:** Figure 14 describes too little of the data. The 16 features described in the text as inputs are not described in detail.

**Comment 5:** For trained models, there is a lack of curves for the iterative process and there is a lack of validation results for the models. It is not possible to prove the actual accuracy of the trained model.

**Comment 6:** Although the article employed three machine learning algorithms for prediction, it fails to explain the reason behind the superior training effect of XGBoost compared to others. Was it due to the amount or type of data, or some other factor?

**Comment 7: **The descriptions of the models for machine learning and the presentation of the results in the paper are too rough. It is recommended to learn about machine learning model drawing and optimize the model schematic. For example, Li *et al.,* (2023) ^1^.

Is the work clearly and accurately presented and does it cite the current literature?

Yes

If applicable, is the statistical analysis and its interpretation appropriate?

Partly

Are all the source data underlying the results available to ensure full reproducibility?

Partly

Is the study design appropriate and is the work technically sound?

Partly

Are the conclusions drawn adequately supported by the results?

Partly

Are sufficient details of methods and analysis provided to allow replication by others?

Yes

Reviewer Expertise:

Intelligent manufacturing

I confirm that I have read this submission and believe that I have an appropriate level of expertise to confirm that it is of an acceptable scientific standard, however I have significant reservations, as outlined above.

## References

[1] : A review of the multi-dimensional application of machine learning to improve the integrated intelligence of laser powder bed fusion. *Journal of Materials Processing Technology* .2023;318: 10.1016/j.jmatprotec.2023.118032 10.1016/j.jmatprotec.2023.118032

# Peer review: Reviewer response for version 1

**DOI:** 10.5256/f1000research.136256.r151891

**Published:** 2022-12-12

**Authors:** Janaki Vinjamuri

**Affiliations:** 1 Department of Computer Science and Engineering, Vaagdevi College of Engineering, Warangal, Telangana, India

Magnesium alloy plays an important role in the automobile industry and understanding its formability is crucial hence the work is much useful. The prediction of formability of magnesium alloy can give a free hand for people in Industry to decide on the usage in different points while building the automobile. The work falls in the category of prediction of materials’ properties hence Machine Learning is a good research area to explore the possible methods and Authors have aptly chosen to implement. The work explored different research contributions and the drawbacks there which paved a path in that direction in defining the problem under consideration. Machine Learning methods to predict Magnesium alloy formability have been experimented using Python. The prediction in this work was done using a proper dataset and for rebuilding by any researchers it is also made openly available. The machine learning models explored includes XGBOOST, Multi Linear Regression and Random Forest which are suitable models for these types of tasks.

 A bit of more detailed discussion on concepts of Random Forest and XGBoost would help other researchers to further explore this kind of problems. The authors tried to demonstrate use of machine learning models in predicting the formability without actually needing to carry out the physical experimentation. The authors considered 7 parameters at 3 different temperatures giving a variety of training examples to train the ML model. The data set used is consisting of 50534 examples is considerably good size dataset for a model to learn the classification pattern. This was successfully demonstrated with the help of afore mentioned models. The results shown are promising and a good detailed discussion is done Random forest is an ensemble method and weighted voting would always leads to a more accurate classification of data. Hence the work can be considered as a good research work which can show some direction for future researchers as well.

Is the work clearly and accurately presented and does it cite the current literature?

Yes

If applicable, is the statistical analysis and its interpretation appropriate?

Yes

Are all the source data underlying the results available to ensure full reproducibility?

Yes

Is the study design appropriate and is the work technically sound?

Yes

Are the conclusions drawn adequately supported by the results?

Yes

Are sufficient details of methods and analysis provided to allow replication by others?

Yes

Reviewer Expertise:

Machine Learning, Deep Learning, Information Security, Network Security

I confirm that I have read this submission and believe that I have an appropriate level of expertise to confirm that it is of an acceptable scientific standard.
